# Unveiling a foreign body masquerading as periarticular calcification: a case report

**DOI:** 10.1186/s13256-024-04475-6

**Published:** 2024-05-14

**Authors:** Amirhossein Kamalinia, Asal Seifaei, Seyed Arman Moein, Hamid Namazi

**Affiliations:** 1 Bone and Joint Diseases Research Center, Shiraz University of Medical Sciences, Shiraz, Iran; 2 Student Research Committee, Shiraz University of Medical Sciences, Shiraz, Iran; 3 Research Center for Non-Communicable Diseases, Jahrom University of Medical Sciences, Jahrom, Iran

**Keywords:** Foreign body, Periarticular calcification, Metacarpophalangeal joint, Case report, Medical history taking, Trauma history

## Abstract

**Introduction:**

Evaluating isolated extremity discomfort can be challenging when initial imaging and exams provide limited information. Though subtle patient history hints often underlie occult pathologies, benign symptoms are frequently miscategorized as idiopathic.

**Case presentation:**

We present a case of retained glass obscuring as acute calcific periarthritis on imaging. A 48-year-old White male with vague fifth metacarpophalangeal joint pain had unrevealing exams, but radiographs showed periarticular calcification concerning inflammation. Surgical exploration unexpectedly revealed an encapsulated glass fragment eroding bone. Further history uncovered a forgotten glass laceration decade prior. The foreign body was removed, resolving symptoms.

**Discussion:**

This case reveals two imperative diagnostic principles for nonspecific extremity pain: (1) advanced imaging lacks specificity to differentiate inflammatory arthropathies from alternate intra-articular processes such as foreign bodies, and (2) obscure patient history questions unearth causal subtleties that direct accurate diagnosis. Though initial scans suggested acute calcific periarthritis, exhaustive revisiting of the patient’s subtle decade-old glass cut proved pivotal in illuminating the underlying driver of symptoms.

**Conclusion:**

Our findings underscore the critical limitations of imaging and the vital role that meticulous history-taking plays in clarifying ambiguous chronic limb presentations. They spotlight the imperative of probing even distant trauma when symptoms seem disconnected from causative events. This case reinforces the comprehensive evaluation of all subtle patient clues as key in illuminating elusive extremity pain etiologies.

## Introduction

Acute calcific periarthritis is an inflammatory condition characterized by calcium hydroxyapatite deposition within periarticular soft tissues, precipitating an intense localized inflammatory response [1]. Notably, even with advanced imaging, differentiating acute calcium deposition diseases from mimickers poses a diagnostic challenge in atypical presentations involving the hands and fingers [2].

Evaluating isolated extremity pain can be challenging when initial exams and imaging provide limited information. Categorizing nonspecific symptoms as idiopathic is often used as a catch-all diagnosis. However, subtle hints in remote patient histories often reveal pain causes [3, 4]. Although, patients may not recall causative trauma, remote injuries can cause foreign body retention, leading to delayed inflammation and discomfort. Thus, a common mistake in assessing chronic limb pain is solely focusing on the site of pain, rather than considering the patient’s complete medical history and any underlying causes [5, 6]. Seemingly minor details buried in a patient’s account may shed light on occult pathologies underlying their complaints. In cases of nonspecific limb discomfort, benign-appearing symptoms may signal retained foreign bodies or other occult disease processes [7–9].

We present a case of retained foreign glass as an unexpected finding in the workup of chronic finger pain despite intact skin and unrevealing initial studies. Sharp hand injuries with foreign bodies are commonly seen in daily orthopedic practice. This case spotlights the imperativeness of re-examining even obscure historical subtleties when evaluating diagnostically evasive extremity pain to illuminate the underlying drivers of patients’ symptoms. This experience serves as a reminder for meticulous history-taking and pre-operative evaluation in orthopedic surgeries.

## Case presentation

**Fig. 1:**
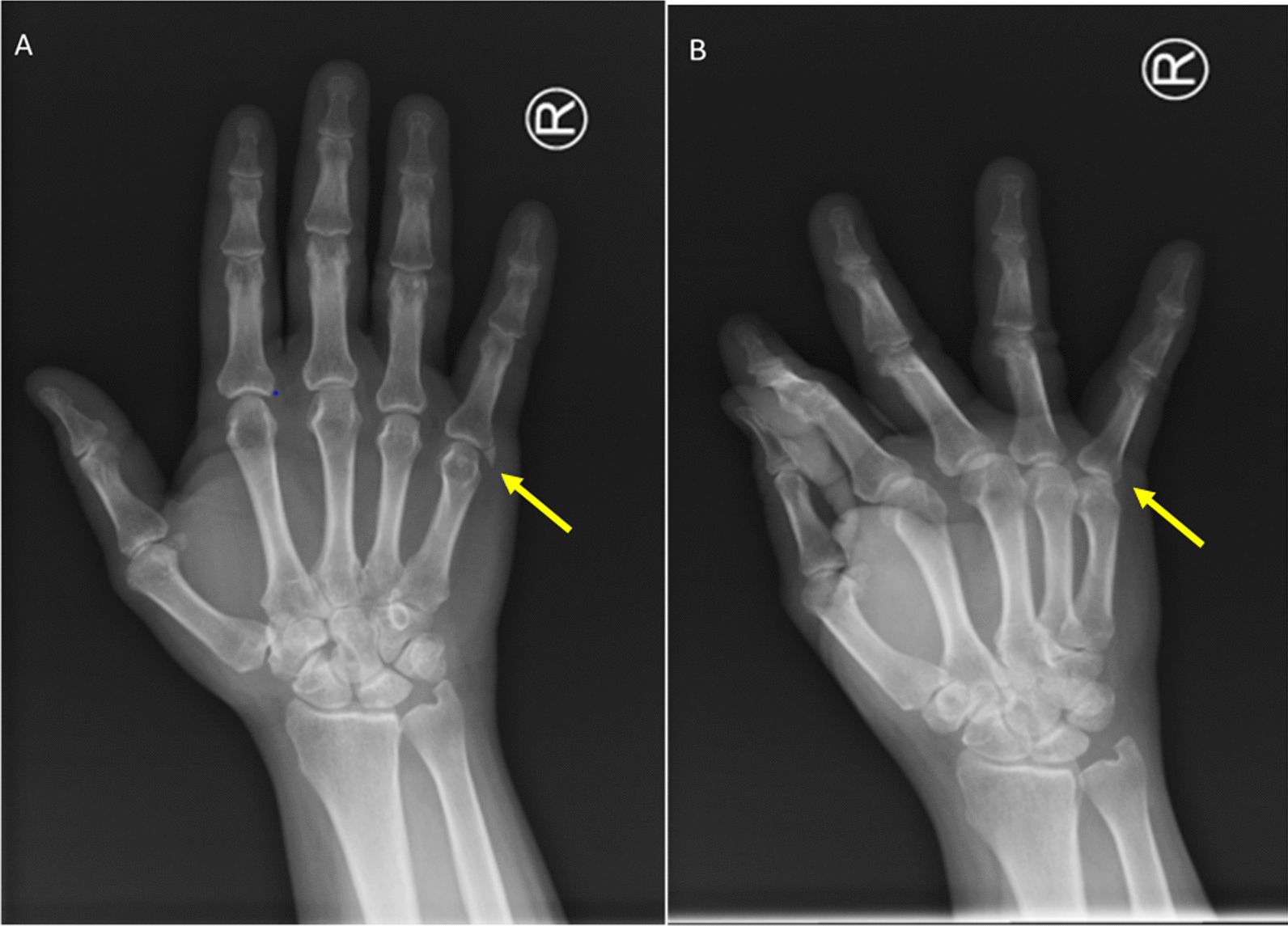
Radiographic imaging showing a calcified lesion concerning for inflammation. **A** Posterior-anterior view of right hand demonstrating calcification (arrow) adjoining ulnar collateral ligament of fifth metacarpophalangeal joint. **B** Oblique view providing additional visualization of periarticular calcification (arrow)

A 48-year-old White male presented to our clinic with a chief complaint of vague and intermittent pain at the ulnar aspect of the fifth metacarpophalangeal (MCP) joint of the right hand. With a blood pressure of 130/80 mmHg, heart rate of 86 beats per minute, respiratory rate of 21 breaths per minute, and temperature of 37.2 °C, vital signs were normal. His past medical history was not remarkable, and he had no familial history of related diseases such as rheumatoid arthritis. He had no recollection of trauma to his hand. The patient’s physical examination revealed mild tenderness to palpation over the ulnar collateral ligament, without appreciable swelling, erythema, ecchymosis, wound, or scar. Range of motion and strength testing were within normal limits. Besides a borderline first-hour erythrocyte sedimentation rate (ESR) 22 mm/hour (normal range 0–20 mm/hour), his lab data was not remarkable. Complete blood count (CBC), C-reactive protein (CRP), urine analysis, and rheumatologic work-ups showed no abnormality. Radiographic evaluation showed a calcified lesion adjoining the ulnar collateral ligament at the fifth MCP joint, indicating possible chronic inflammation (Fig. 1). Initially, with a possible diagnosis of chronic calcified peroarthritis and inflammation of the ulnar collateral ligament of the fifth MCP joint, medical management was chosen. Acetaminophen tablet 500 mg three times per day and indometacin tablet 75 mg two times per day were prescribed. Yet, the patient’s symptoms did not diminish with medical therapy.

**Fig. 2:**
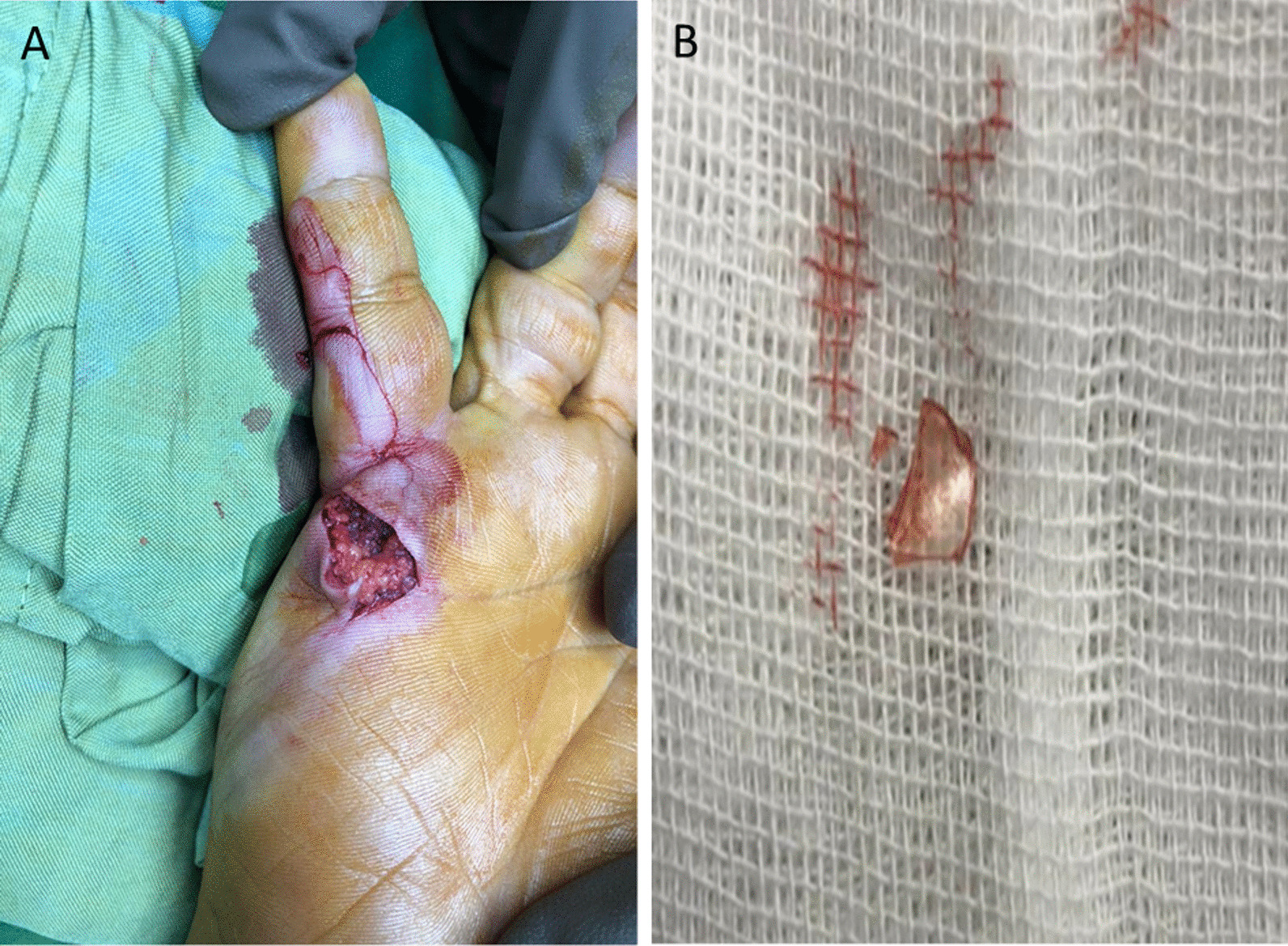
Discovery of glass foreign body. **A** Intraoperative exposure revealing an unexpected finding (not shown). **B** Retrieved encapsulated glass fragment eroding into bone (not pictured)

Given the uncertainty regarding overall diagnostic accuracy, the patient was recommended for surgical intervention for further management of the underlying pathology. During the surgical exploration, a glass foreign body encapsulated in fibrotic tissue was identified, eroding into the underlying bone of the fifth MCP joint (Fig. 2). Upon further history-taking postoperatively, the patient recalled sustaining an unreported glass laceration to the volar right hand nearly 10 years prior, for which he did not seek medical attention given the lack of symptoms at the time. Apparently, the injury had left no visible scar. He was discharged the same day with no problem regarding the surgery.

**Fig. 3:**
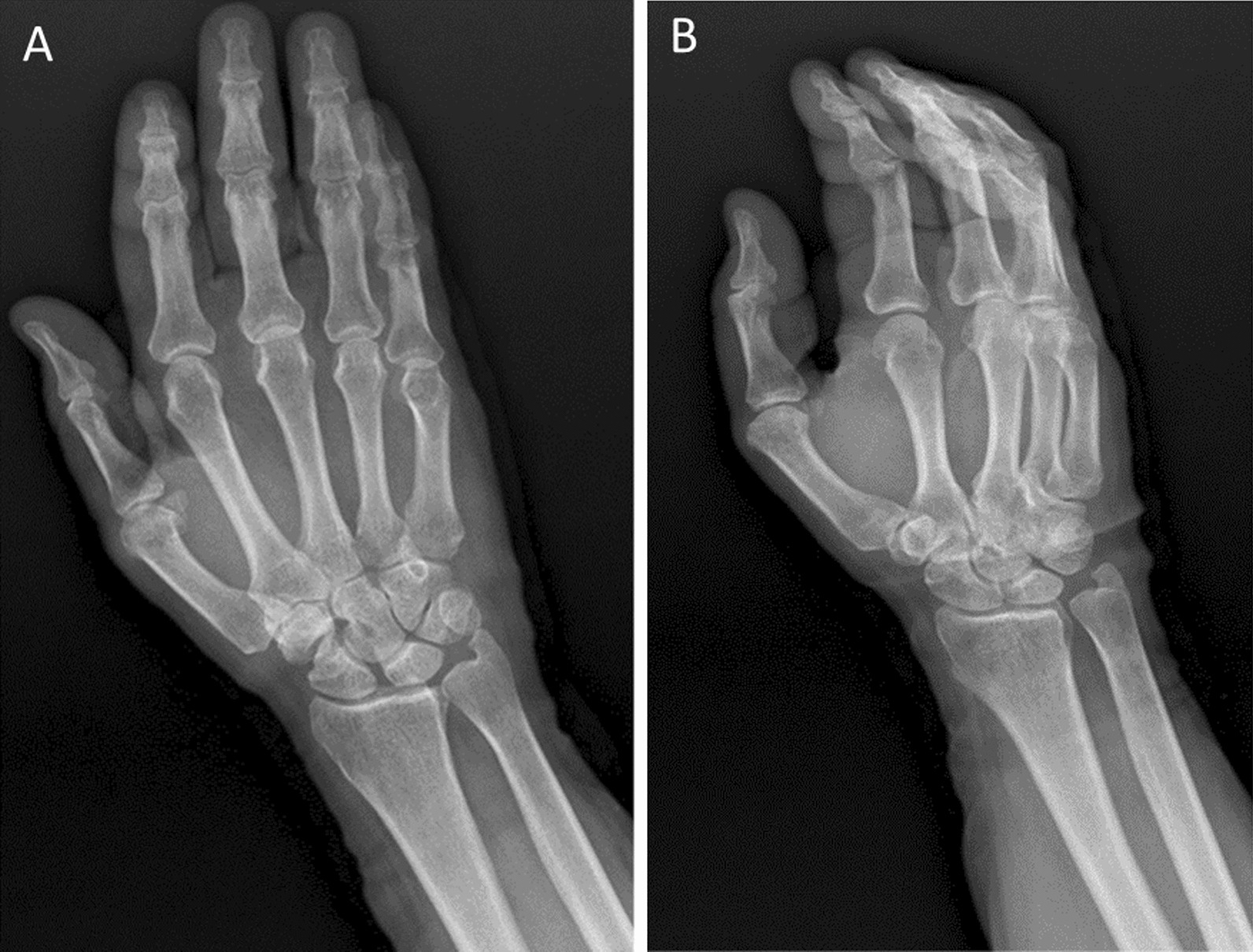
Postoperative imaging after glass removal. **A** Posterior-anterior hand radiograph showing interval removal of the calcified lesion. **B** Oblique perspective demonstrating postsurgical changes at the fifth metacarpophalangeal joint space after glass foreign body removal

The patient had an uncomplicated postoperative course with complete symptom resolution at 6-week follow-up (Fig. 3). Furthermore, the patient had no complaint regarding his surgery and had no complication in his finger function at 6-month follow-up.

## Discussion

In this article, we present a case of chronic fifth metacarpophalangeal joint pain. Though radiographs showed a calcific lesion, surgical exploration revealed an encapsulated glass foreign body, an unexpected decade-old relic the patient had forgotten until the postoperative history re-taking highlighted the vital value of comprehensively probing even obscure historical clues when evaluating vague limb pain.

A complete medical history is a vital component of patient evaluation, and yet subtleties may be overlooked without iteratively revisiting with directed questioning. Patients frequently fail to associate remote or seemingly insignificant injuries with current symptoms, diminishing perceived relevance [4, 6, 10].

In a similar case, a 21-year-old female with progressive foot pain had a lesion resembling a vascular malformation. Finally, during the surgery, it turned out that it was a wood splinter in her foot, and she did not recall an event of stepping on a broken broomstick [11].

However, obscurities uncovered on careful historical re-examination frequently illuminate explanatory pathology. In cases of nonspecific extremity discomfort such as this one, exhaustive recounting of past trauma, even minor lacerations healed without intervention, is imperative [7, 8]. Focused history-taking is equally essential at postoperative follow-ups when new symptom context guides identification of relevance in previous events [12]. As this case reveals, a forgotten glass cut causing no initial issues precipitated future complications necessitating eventual surgery. The dramatic symptom resolution post-extraction demonstrates how remote subtleties direct diagnostic accuracy in perplexing presentations. This requires thorough interviewing and analysis of all potential triggering factors to achieve a comprehensive diagnosis.

Also, several inflammatory arthropathies can present similarly to foreign body retention on preliminary imaging. Foreign material may mimic hydroxyapatite deposition disease, though less common in the hand, and will demonstrate calcific focus with surrounding inflammation [13, 14]. Meanwhile, embedded fragments can observationally overlap with gout and pseudogout frequently which manifests as erosions with sclerotic margins and soft tissue swelling [15]. Even synovial osteochondromatosis, though distinguishable by loose intra-articular bodies, causes bony projections potentially confused with external debris [16]. These considerations showcase how advanced imaging lacks the specificity to accurately diagnose intra-articular pathology in atypical presentations [17]. While sensitive for joint disease, areas of calcification or ossification cannot delineate if they result from endogenous pathophysiology versus exogenous introduction. This limitation underscores the vital value of history-taking in diagnostically clarifying ambiguous scans [18, 19].

## Conclusion

In conclusion, our case report enhances overall comprehension of such cases and reinforces the importance of a comprehensive approach in diagnosing and managing similar conditions. Our findings underscore the critical significance of thorough history-taking in uncovering latent musculoskeletal etiologies, emphasizing the pivotal role this aspect plays in clinical practice.

## Data Availability

Data of the patient can be requested from authors. Please write to the corresponding author if you are interested in such data.

## References

[1] Doumas C, Vazirani RM, Clifford PD, Owens P (2007). Acute calcific periarthritis of the hand and wrist: a series and review of the literature. Emerg Radiol.

[2] Tomori Y, Nanno M, Takai S (2020). Acute calcific periarthritis of the proximal phalangeal joint on the fifth finger: a case report and literature review. Medicine.

[3] Kent P, Keating JL (2005). Classification in nonspecific low back pain: what methods do primary care clinicians currently use?. Spine.

[4] Goldberg DS (2017). Pain, objectivity and history: understanding pain stigma. Med Humanit.

[5] Hannoodee S, Nasuruddin DN. Acute inflammatory response. 2020. 32310543

[6] Carneiro BC, Cruz IA, Chemin RN, Rizzetto TA, Guimarães JB, Silva FD (2020). Multimodality imaging of foreign bodies: new insights into old challenges. Radiographics.

[7] Lea E, Nawaf H, Yoav T, Elvin S, Ze’ev Z, Amir K (2005). Diagnostic evaluation of foreign body aspiration in children: a prospective study. J Pediatr Surg.

[8] Ploner M, Gardetto A, Ploner F, Scharl M, Shoap S, Bäcker H (2020). Foreign rectal body—systematic review and meta-analysis. Acta Gastro-Enterol Belg.

[9] Owjfard M, Karimi F, Mallahzadeh A, Nabavizadeh SA, Namavar MR, Saadi MI (2023). Mechanism of action and therapeutic potential of dimethyl fumarate in ischemic stroke. J Neurosci Res.

[10] Nabavizadeh SA, Khorraminejad-Shirazi M, Firouzabadi D, Nabavizadeh SS, Jafari SH, Dehghanian A (2023). Osteoma in the upper cervical spine: a case report and comprehensive literature review. Int J Surg Case Rep.

[11] Armstrong D, Owens R, Carlson T, Ching B (2024). Retained foreign body misdiagnosed as a low flow vascular malformation: a case report. Radiol Case Rep.

[12] Peart P (2022). Clinical history taking. Clin Integr Care.

[13] Hongsmatip P, Cheng KY, Kim C, Lawrence DA, Rivera R, Smitaman E (2019). Calcium hydroxyapatite deposition disease: imaging features and presentations mimicking other pathologies. Eur J Radiol.

[14] Benvenuto P, Locas S (2020). Hydroxyapatite deposition disease: a common disease in an uncommon location. Intern Emerg Med.

[15] Gudapati O (2021). 16 Crystal deposition disorders. Textbook of orthopedic rheumatology.

[16] Abakka F (2021). Imaging of primary synovial osteochondromatosis: a case report. Sch J Med Case Rep.

[17] Park EH, Fritz J (2023). The role of imaging in osteoarthritis. Best Pract Res Clin Rheumatol.

[18] Yan JF, Qin WP, Xiao BC, Wan QQ, Tay FR, Niu LN (2020). Pathological calcification in osteoarthritis: an outcome or a disease initiator?. Biol Rev.

[19] Lai Y, Yu R, Hartwell HJ, Moeller BC, Bodnar WM, Swenberg JA (2016). Measurement of endogenous versus exogenous formaldehyde-induced DNA-protein crosslinks in animal tissues by stable isotope labeling and ultrasensitive mass spectrometry. Cancer Res.

